# Contralateral Risk-Reducing Mastectomy: Review of Risk Factors and Risk-Reducing Strategies

**DOI:** 10.1155/2015/901046

**Published:** 2015-01-27

**Authors:** N. N. Basu, L. Barr, G. L. Ross, D. G. Evans

**Affiliations:** ^1^Nightingale and Genesis Prevention Centre, University Hospital South Manchester, Southmoor Road, Manchester M23 9LT, UK; ^2^Department of Breast Surgery, Queen Elizabeth Hospital, Birmingham B15 2TH, UK; ^3^The Institute of Cancer Sciences, The University of Manchester, Oxford Road, Manchester M13 9PL, UK; ^4^University of Manchester Department of Genomic Medicine, Institute of Human Development, St. Mary's Hospital, Oxford Road, Manchester M13 9WL, UK

## Abstract

Rates of contralateral risk-reducing mastectomy have increased substantially over the last decade. Surgical oncologists are often in the frontline, dealing with requests for this procedure. This paper reviews the current evidence base regarding contralateral breast cancer, assesses the various risk-reducing strategies, and evaluates the cost-effectiveness of contralateral risk-reducing mastectomy.

## 1. Introduction

Breast cancer is the most common cancer in women worldwide, with 1.7 million new cases diagnosed in 2012 [1, 2]. It accounts for 25% of all cancers in women and in the UK; it is estimated that 1 in 8–10 women will develop breast cancer [3] during their lifetime.

Breast cancer survivorship has improved as a result of early detection and advancing treatment modalities [3]. As such, management of this group of women requires healthcare professionals to be familiar with additional risks factors so that timely recommendations may be made on surveillance or risk-reducting strategies.

Once diagnosed with breast cancer, these women will have an increased risk of developing a contralateral, metachronous breast cancer [4]. The level of this risk is multifactorial, dependent on tumour biology, adjuvant treatment, and oncogenetics.

There is ongoing interest in contralateral breast cancer (CBC). Surveillance, Epidemiology, and End Results Program (SEER) data from the US [5] has confirmed a 150% increase in rates of contralateral risk-reducing mastectomy (CRRM) over the last decade, although this may not be the case in Europe [6]. This rise is somewhat surprising, given that rates of CBC are decreasing (due to endocrine therapy especially with aromatase inhibitors) and may be a reflection of a heightened perceived risk in this vulnerable group of women. Clinicians are at the frontline of many of these complex decisions and need to make evidence-based recommendations.

We examine the multiple risk factors known to contribute to developing CBC. Survival data are analysed and the contribution of the following risk factors is discussed: gene mutation and family history, histology, ER status, and HER2 status. We evaluate the different risk-reducing strategies (surgery and chemoprevention) and their efficacy and cost and finally consider the patient's perspective. We have included chemoprevention using antiendocrine treatment in the section of ER status as this is relevant to this section.

This review aims to serve as an aide-memoire for clinicians to refer to when counselling women on CBC.

## 2. Incidence of CBC

High-risk patients include breast cancer sufferers with known genetic mutations (*BRCA1/2, TP53*) and a significant family history.* BRCA1/2* mutation carriers have a CBC risk of 2-3% per annum [8]. This is likely to be higher in* TP53* patients, though there is limited data on this group of patients [9]. This heightened risk, particularly in women diagnosed with their primary cancer before 40 years, lasts for at least 20 years. Risk-reducing bilateral salpingo-oophorectomy (RRBSO) and menopause before the age of 40 [8, 10, 11] are protective factors.

The remainder of patients without a known genetic mutation or significant family history represent the majority of breast cancer sufferers. SEER historical data (1973–1996) quote actuarial incidence rates of CBC in this group of women at 5, 10, 15, and 20 years were 3%, 6.1%, 9.1%, and 12%, respectively, amounting to 0.6% per annum [4]. This level of risk is likely to be outdated with several studies supporting a global decrease in CBC, with almost a 30% reduction in parts of Europe over the last 10-year period [4]. This trend is likely to be a reflection of successful adjuvant treatment, in particular antiendocrine treatment.

### 2.1. Risk Factors

#### 2.1.1. Gene Mutation

The two most commonly studied breast cancer susceptibility genes are* BRCA1* and* BRCA2*. Mutations in these tumour suppressor genes confer an up to 80–90% lifetime risk of developing breast cancer [12–14].

These women have a significant CBC risk once diagnosed with breast cancer.

The risk, up to age 70 years, of a CBC in* BRCA1* mutation carriers has been estimated to be above 60% [15] and in* BRCA2* mutation carriers slightly lower at around 50% [12]. However, a recent prospective study [16] has shown that these risks may be even higher at 83% and 63%, respectively, representing higher risks in the modern era particularly for* BRCA1* where the majority of tumours would not receive endocrine therapy.

Several studies have evaluated further CBC risk factors within* BRCA1/2* mutation carriers [8, 10, 11]. Early age of first breast cancer diagnosis (<50 years) with increasing numbers of first-degree relatives with breast cancer at a young age heightens that risk. Protective factors that reduce CBC risk include the use of tamoxifen (HR 0.59; 95% CI 0.31–1.01) [8, 17] and oophorectomy (HR 0.44; 95% CI 0.21–0.91), with additional benefit of oophorectomy prior to the age of 49 years (HR 0.24; 95% CI 0.07–0.77).

Breast cancer remains the most common malignancy in women harbouring a* TP53 *mutation in Li-Fraumeni syndrome. These mutation carriers will have a nearly 100% lifetime risk of developing cancer with the vast majority developing breast cancer by the age of 46 [18].

Given the rarity of this condition, there is very limited data on CBC risk. Evans et al. [9] studied women under the age of 30 years with breast cancer and found that rates of CBC were approximately 2-3% annually in all mutations carriers (*TP53* and* BRCA1/2*), although only 11* TP53* mutation carriers were included in their extended analysis. It is possible that adjuvant endocrine and anti-HER2 treatment will influence CBC risk in* TP53* mutation carriers as the majority of these patients are ER and HER2 positive [19]. Efforts are underway to assess this risk further.

#### 2.1.2. Family History

A positive family history of breast cancer increases the risk of CBC although this risk pattern is complex. Vichapat et al. [7] studied 8478 women with breast cancer over a 31-year period (1975–2006) and found that there was a 2.8-fold increase in relative risk with a positive family history of breast cancer. Subgroup analysis revealed that the highest risks were those with a first- and second-degree relative (RR 2.33) followed by first-degree alone (RR 1.38) and second- or third-degree (RR 1.13). Numerous first-degree relatives will confer an even higher risk.

The WECARE study (Women's Environment Cancer and Radiation Epidemiology Study) conducted a population-based case control study comparing asynchronous bilateral breast cancer patients (case) against unilateral breast cancer (control) patients [20]. They confirmed that the risk of CBC in noncarriers of* BRCA1/2* mutation with a family history was highest in women diagnosed at an earlier age with their index breast cancer (<45 years), those with a young first-degree relative, particularly with bilateral disease. The 10-year cumulative CBC risk stratified by age was 6.7% (50–54 years), 9.0% (40–44 years), and 14.7% (30–34 years).

A study from the Mayo Clinic [21] followed up 745 women with breast cancer and a positive family history who underwent a CRRM between 1960 and 1993. They had predicted (without CRRM) 106 CBCs in the premenopausal group and 50 CBCs in the postmenopausal group. CRRM had resulted in an approximate 95% reduction in relative risk as only 6 and 2 actual CBCs occurred in pre- and postmenopausal women, respectively.

#### 2.1.3. Histology of Index Breast Cancer

The Vichapat et al. study [7] assessed histological type and found no significant increase in CBC in those with lobular breast cancer. This is an interesting finding given that lobular breast cancer has been shown previously to be an independent predictor of increased CRRM rates [22] and possibly arises from previous studies that have shown an association of lobular cancer and CBC [23].

High grade of primary tumours (RR 1.3 for Grade 3 cancer compared to Grade 1), increasing size (<2 cm RR 1.0, 2–5 cm RR 1.51, and >5 cm RR 1.89), and number of positive lymph nodes (non-RR 1.0, 4–9 RR 1.12, and >10 RR 1.62) were all shown to be important risk factors [7].  Table 1 summarises the known risk factors for developing contralateral breast cancer while Table 2 shows the risk reduction strategies of CBC.

#### 2.1.4. HER2 Status and Anti-HER2 Treatment

Up to 30% of breast cancers [24] express HER2 receptor tyrosine-protein kinase. Use of the monoclonal antibody trastuzumab (Herceptin) has been shown to improve disease-free survival [25]. The HERA study (Herceptin Adjuvant Trial) recently reported outcomes after a 4-year follow-up [26]. In the observation group there were 19/320 (1.1%) CBCs compared to 14/251 (0.8%) in the trastuzumab group. Although this represents a small reduction in CBC in women treated with trastuzumab, its clinical application for risk reduction of CBC remains debatable.

Saltzman et al. [27] performed a case-control study of 29,126 women using the Cancer Surveillance System (CSS) cancer registry. They were able to show that women with HER2 overexpression (ER negative/HER2 positive) and those with triple negative cancer (ER negative, PR negative, and HER2 negative) had a 2.0-fold and 1.4-fold increased risk of developing CBC, respectively. Therefore, in addition to having a higher risk of recurrent disease and death, this subgroup of patients will have an elevated risk of CBC and surveillance strategies need to be considered in monitoring this cohort.

#### 2.1.5. ER Status and Chemoprevention

Several studies (ATAC, IBIS I, IBIS II, and STAR) have confirmed that antihormonal agents (SERM and aromatase inhibitors) given to high-risk women for up to 10 years can reduce the incidence of CBC and primary breast cancer [28]. Women with hormone sensitive index breast cancers are routinely offered antihormonal agents as part of their adjuvant treatment and can expect an up to 50% reduction in their risk of developing a CBC with recent studies showing favouring aromatase inhibitors over tamoxifen (ATAC) in the postmenopausal setting [29]. Women considering antihormonal agents need to be appraised of significant adverse effects including thromboembolic phenomenon, osteoporosis, and uterine carcinoma.

Recently, Gronwald et al. [17] were able to confirm previous studies showing an approximately 50% reduction in CBC risk in* BRCA1/2* mutation carriers who took Tamoxifen following their index breast cancer. Of interest was the similar risk-reduction of a short period of Tamoxifen (<1 year) compared to longer use (>4 years). This has implications on women who have concerns over the side effect profile of long term Tamoxifen use and may rationalise the short-term use of this drug.

#### 2.1.6. Chemotherapy

Cytotoxic chemotherapy agent recommended as adjuvant or neoadjuvant treatment of primary breast cancer has been shown to reduce the risk of CBC. The Early Breast Cancer Trialists Group (EBCTG) showed a marginal reduction in the incidence of CBC over a 15-year follow-up period [30], which was more definite in women under the age of 50 years.

The WECARE group carried out a case-control study [31] and found that chemotherapy use was associated with a 35–40% reduction in risk of CBC in women under the age of 55 and that this protective effect lasted up to 10 years. In addition, those who became postmenopausal within 1 year of diagnosis had the greatest risk reduction.

PARP inhibitors and their effect on triple negative and* BRCA1/2 *mutation related breast cancers are the subject of much interest [32]. Initial reports from proof-of-concept trials [33] have confirmed their safety and efficacy, and phase III studies with an extended follow-up may determine whether these targeted therapy modalities affect contralateral breast cancer risk.

### 2.2. Contralateral Risk-Reducing Mastectomy

#### 2.2.1. Survival

The aftermath of Angelina Jolie's announcement of her bilateral risk-reducing mastectomy (BRRM) has raised public awareness of risk-reducing surgery [34, 35]. Several studies have confirmed a survival benefit in high-risk patients (*BRCA1/2* and FH) undergoing BRRM and CRRM [36–39]. Our own experience [39] compared 105 female* BRCA1/2* mutation carriers with unilateral breast cancer to matched mutation carriers who did not undergo CRRM. The overall 10-year survival was 89% in the CRRM group compared to 71% in the non-CRRM group (*P* < 0.001), which was independent of RRBSO. This is in contrast to van Sprundel et al. [40] who found that, after adjusting for RRBSO, there was no overall survival benefit from CRRM. Metcalfe et al. [38] followed a similar group of 390* BRCA1/2* mutation carriers and found that at 20 years the survival rate of those who underwent CRRM was 88% (CI 83–93%) compared to 65% (CI 59–73%) who did not undergo CRRM.

The survival benefit in non-*BRCA* mutation patients is less clear. Younger women under the age of 49 [41, 42] with ER −ve disease are likely to have an improved disease-specific survival, which is thought to be due to a higher baseline risk of CBC. A clear survival benefit on the remainder of patients seems less likely [37]. Portschy et al. [43] used a Markov model to compare survival outcomes between CRRM and non-CRRM in non-*BRCA* patients. They estimated a less than 1% absolute 20-year survival benefit from CRRM amongst all age groups, ER status, and cancer stage groups.

#### 2.2.2. Breast Reconstruction

Several studies have shown that access to immediate breast reconstruction positively affects the decision for CRRM [44, 45]. Recently, Ashfaq et al. [45] identified 102,674 patients (2004–2008) with a diagnosis of DCIS (15%) or invasive breast cancer (85%) from SEER registry data. Those undergoing mastectomy were 3 times more likely to request CRRM if offered immediate reconstruction. Overall, 16% of all patients underwent CRRM with a significant proportion undergoing reconstruction (46%, *P* < 0.001). Similar proportions of patients underwent implant-based reconstruction (36%) and tissue-based reconstruction (37%). There was a trend of increasing numbers of reconstructions during this time period, and Caucasian women, under the age of 45 years with a diagnosis of a node-negative lobular carcinoma or DCIS, were more likely to choose reconstruction.

Women undergoing reconstruction following CRRM are 1.5 times more likely to have a major complication requiring hospitalisation or reoperation [46] compared to unilateral mastectomy. Limited data is available comparing CRRM and reconstruction with unilateral mastectomy and reconstruction. Crosby et al. [47] assessed 497 patients undergoing CRRM with reconstruction and concluded that a third of patients experiencing at least one complication may not have developed a complication if they had only had a mastectomy and reconstruction of their index side.

#### 2.2.3. Sentinel Lymph Node Biopsy (SLNB)

SLNB at the time of risk-reducing surgery remains controversial. A recent meta-analysis of 1251 patients [48] showed that 1.7% (*n* = 21) of women undergoing RRM harboured occult invasive cancer in the mastectomy specimen. Of these 21 patients, the SLN was positive in only 4/21 patients and negative in the remainder (17/21). This was higher in women with advanced cancer in the contralateral breast. Overall, 2.8% (*n* = 36) of women benefited from SLNB that included 19 cases of a positive SLNB result requiring completion axillary surgery and 17 women who had invasive disease in the mastectomy specimen but a negative SLNB, thus avoiding further axillary surgery. This is offset against the 5% lymphoedema rate associated with SLNB [49]. Kuwajerwala et al. [50] retrospectively assessed 170 patients undergoing CRRM and found that of the 21.8% who had a SLNB at the surgeon's discretion, none had positive SLNB.

#### 2.2.4. Cost

Health care economics contribute to the decision making process. Cost-effectiveness with life expectancy gains is well established in the setting of bilateral RRM in women harbouring* BRCA1/2* mutations [51, 52]. Few studies have looked at this in the setting of CRRM [53–55]. Deshmukh et al. [54] analysed matched groups (CRRM and non-CRRM) and showed that CRRM significantly increases short-term healthcare cost by $7,749. In addition, women who had a reconstruction and in particular a delayed type had significantly higher cost associated as well as those who had HER2 positive disease and received radiotherapy.

In contrast, Roberts et al. [55] found that CRRM was cost-effective in the prevention of CBC in women under the age of 50 years. From their decision tree-model, they concluded that 68,000 women under the age of 50 years would have been diagnosed with early breast cancer in 2010. If all women had undergone CRRM, savings of $19 million would have been made to avoid 3,900 contralateral breast cancers that would have developed over the next 10 years. Their CRRM group had 0.2 quality-adjusted life years (QUALYs) less than the non-CRRM that may have been accounted for by complications of reconstruction. They highlighted a potential greater benefit of CRRM in ER −ve disease compared to ER +ve disease, given that the latter would receive adjuvant endocrine treatment, shown to reduce CBC.

Zendejas et al. [53] used a Markov model to compare cost-effectiveness in women undergoing CRRM compared to routine surveillance (including annual mammography). They found that CRRM prior to the age of 70 years was cost-effective and in particular in those who were BRCA-positive.

Currently in the UK, there are no funding restrictions within the National Health Service on CRRM. Breast cancer patients can choose between delayed and immediate (performed at the same time as the therapeutic mastectomy) without financial scrutiny provided that there is backing from the relevant clinicians.

#### 2.2.5. Patient's Perspective

One of the main driving forces for CRRM is patients' worry and anxiety about developing another breast cancer and having to undergo further treatment including chemotherapy. This is often the most difficult component to assess, as the psychology behind it is multifactorial. A recent US study reported that 68.9% of patients undergoing CRRM did not have genetic or familial risk factors for CBC [56] and that the main driving force for this was worry about recurrence. Patients overestimate their risk of contralateral breast cancer [57] and in doing so can compound their anxiety.

A recent study [58] assessed the perspective from 60 consecutive patients choosing CRRM. In almost all cases, requests for CRRM were instigated by the patient and every patient unambiguously wanted CRRM. Patients responded to risk in an “all or nothing” manner and the majority did not objectively quantify this risk. The risk assessment in those that did quantify risk had little role in their decision for surgery. The authors concluded that “patients' subjective sense of vulnerability overwhelmed their appreciation of risk so that, so that regardless of level of risk of CBC, they found this risk intolerable and felt that only CRRM could reduce it.” This showed that a rate limiting factor will be the availability of immediate reconstruction and if this is not made possible for patients as part of their primary surgical treatment rates of CRRM are likely to be lower.

## 3. Discussion

Rates of CRRM have increased in the US, a trend that in the future other countries may follow. This is of concern given that the actual incidence on CBC is on the decrease as a result of successful adjuvant treatments.

CBC risk assessment is multifactorial and may be assessed in a multidisciplinary setting. The most significant risk factors are gene mutation status and significant family history, which can result in at least a fourfold increase in CBC risk. Patients harbouring a* BRCA1/2* mutation have an approximate 2-3% annual incidence on developing CBC. In nonmutation carriers with a family history, young age at first diagnosis and first-degree relative are particularly strong risk factors.

Tumour biology is important. The ER status is of particular importance given that approximately 70% of all breast cancers are hormone sensitive. Risk reduction with antiendocrine treatment is approximately 50% with tamoxifen and 70% with aromatase inhibitors. As predicted, women with ER negative breast cancer have an increased risk of CBC. The use of cytotoxic agents and targeted treatments (e.g., Herceptin) marginally reduces CBC, with the greatest benefit for young women having chemotherapy.

CRRM offers the greatest risk reduction of CBC, up to 95% in women with a family history. Survival benefits are conferred on those high-risk patients with a* BRCA1/2* mutation. There is no survival benefit in the non-high-risk group.

Access to immediate breast reconstruction positively affects a woman's decision to opt for CRRM, with a significant number experiencing operative complications. Occult disease in the CRRM specimen occurs in less than 2% of women with no clear evidence to support sentinel lymph node biopsy.

Survival benefits and cost-effectiveness are seen in those at the highest risk of CBC (gene mutation carriers) and these patients are likely to benefit most from CRRM.

The assessment of CBC risk is multifactorial and may be assessed in a multidisciplinary setting. Arrington et al. [22] showed that surgeon and patient characteristics determine CRRM and include independent factors like young patient age (<40 years), large tumour size (>5 cm), lobular histology, positive family history, multicentric disease, and female surgeon. In addition, patients' anxiety about developing another breast cancer and going through subsequent treatment is a real entity, albeit difficult to quantify.

All clinicians treating breast cancer patients should be familiar with CBC risk and have the opportunity to discuss the various options including CRRM, chemoprevention, and routine surveillance. The multidisciplinary team is invaluable in guiding women based on objective assessment of genetic and family history, tumour biology, and psychological support.

It is becoming apparent that women seeking CRRM are categorised into different risk groups [59] and that clinicians are faced with differing challenges when managing these different groups. This review has focused on the different risk factors and risk-reducing strategies to give clinicians a comprehensive overview of the current literature.

## Figures and Tables

**Table 1:** Risk factors for developing CBC (estimated annual risk) [4, 7].

	Estimated annual risk (%) [4]	Relative risk—multivariate (95% CI) [7]
*Patient factors *		
Age at first diagnosis		
<30 yrs	0.5–1.3	
40–50 yrs		
ER +ve	0.2-0.3	
ER −ve	0.4-0.5	
Gene mutation		
*BRCA1 *	2.0–3.0	
*BRCA2 *	2.0–3.0	
Family history		
None		1 (reference)
First- and second-degree	0.4–1.3	2.8 (1.4–5.5)
First-degree	0.2–0.8	1.4 (0.9–2.1)
Second- or third-degree	Baseline	1.1 (0.7–1.9)
*Tumour factors *		
Size		
<2 cm (T1)		1 (reference)
2–5 cm (T2)		1.5 (1.1–2.0)
>5 cm (T3)		1.9 (1.1–3.3)
LN status		
None		1 (reference)
1–3		0.9 (0.6–1.2)
4–9		1.1 (0.7–1.9)
>10		1.6 (0.8–3.1)
Histology		
Ductal		1 (reference)
Lobular		1.2 (0.6–2.1)
ER status		
ER positive		1.0 (reference)
ER negative		1.3 (0.9–1.9)
HER2 status		
HER2 positive		1.0 (reference)
HER2 negative		1.02 (0.6–1.8)

**Table 2:** Risk reduction of CBC associated with chemoprevention and surgery.

	Risk reduction (95% CI)
*Chemoprevention *	
Antiendocrine	
Tamoxifen in *BRCA1/2* mutation carriers	OR 0.5 (0.3–0.9)
Tamoxifen in noncarriers	50% risk reduction
Aromatase inhibitors in noncarriers	70% risk reduction
Chemotherapy	
Chemo versus no chemo	RR 0.6 (0.4–0.8)
*Surgery *	20-year survival benefit
CRRM	
CRRM in *BRCA1/2* mutation carriers	14.9%
CRRM in nonmutation carriers	<1%
